# LMO2 promotes tumor cell invasion and metastasis in basal-type breast cancer by altering actin cytoskeleton remodeling

**DOI:** 10.18632/oncotarget.13434

**Published:** 2016-11-17

**Authors:** Ye Liu, Zhaoyang Wang, Di Huang, Chao Wu, Huihui Li, Xin Zhang, Bin Meng, Zongjin Li, Tianhui Zhu, Shuang Yang, Wei Sun

**Affiliations:** ^1^ Laboratory of Molecular Genetics in School of Medicine, Nankai University, Tianjin, China; ^2^ Department of Histology and Embryology in School of Medicine, Nankai University, Tianjin, China; ^3^ Department of Pathology, Tianjin Medical University Cancer Institute and Hospital, Tianjin, China; ^4^ Laboratory of Stem cells in School of Medicine, Nankai University, Tianjin, China

**Keywords:** LMO2, actin filament, basal-type breast cancer, metastasis, cofilin1

## Abstract

LMO2 is traditionally recognized as a pivotal transcriptional regulator during embryonic hematopoiesis and angionenesis, and its ectopic expression in T lymphocyte progenitors is closely correlated to the onset of acute T lymphocytic leukemia. However, recently studies revealed complicated expression features and dual functions of LMO2 on tumor behaviors in a variety of cancer types, including breast cancers. Basal-type breast cancer is one of the breast cancer subtypes and a prognostically unfavorable subtype among all breast cancers. Herein we found that in basal-type breast cancer specifically, high LMO2 expression was positively correlated with lymph node metastases in patients, promoted tumor cell migration and invasion and increased distant metastasis in SCID mice. Moreover, the novel function of LMO2 was achieved by its predominantly cytoplasmic location and interaction with cofilin1, which is a critical regulator in actin cytoskeleton dynamics. These findings suggest a subtype-dependent role of LMO2 in breast cancers and the potential of LMO2 as a subtype-specific biomarker for clinical practice.

## INTRODUCTION

The human *lmo2* gene was first cloned from an acute T lymphocytic leukemia (T-ALL) patient [1], primarily promotes embryonic hematopoiesis and angiogenesis [2–4], and specifically triggers T cell leukemia when ectopically expressed in T cell progenitors [5–7]. Traditionally, LMO2 was recognized as a transcription factor located primarily in cell nuclei in hematopoietic cells and vascular endothelia, and performed bi-directionally regulation functions on its different target genes [8–10]. Interestingly however, the LMO2 protein consists of only two tandem LIM domains which mediate protein-proteins interactions, so it lacks the directly DNA-binding ability and functions as a bridge molecular in the transcriptional complex [11, 12]. Notably, recent studies revealed that LMO2 was expressed in a variety of normal tissues and cancer cells, with either nuclear or cytoplasmic location [13]. Moreover, LMO2 showed complicated expression features in different cancer types and dual functions on tumor behaviors. The expression of LMO2 was increased in low grade glioblastoma, whereas decreased in head and neck, lung, colorectal, breast, renal, uterine corpus endometrioid, and cervical carcinomas compared with their relevant normal tissues [14]. Meanwhile, some reports indicated that LMO2 played an oncogenic role in glioblastoma [15] and prostate carcinoma [16], but was a good prognostic marker for diffuse large B cell lymphoma (DLBCL) [17–19], acute B lymphocytic leukemia (B-ALL) [20] and pancreatic carcinoma [21].

The breast cancer is a kind of highly heterogeneous disease with diverse biological and clinical characteristics. Based on gene expression feature, breast cancers can be subdivided into luminal A, luminal B, Her2, and basal subtypes (the PAM50 subtyping system) [22, 23]. In breast cancers, LMO2 showed an ability of attenuating the canonical Wnt-β-catenin pathway via binding with dishevelled-2 protein in a subtype-independent manner, suggesting a general tumor suppressor role, particularly during the early stage of tumorigenesis [14]. However, further analysis revealed that LMO2 played additionally divergent functions in different breast cancer subtypes. Herein our data supported that specifically in basal type breast cancer, LMO2 played a function of promoting tumor cell migration, invasion and metastasis, and this function was achieved by its cytoplasmic location and blocking effect on LIM kinase 1 (LIMK1)-mediated phosphorylation of cofilin1.

## RESULTS

### High LMO2 expression is positively associated with lymph node metastases in basal-type breast cancer

**Figure 1:**
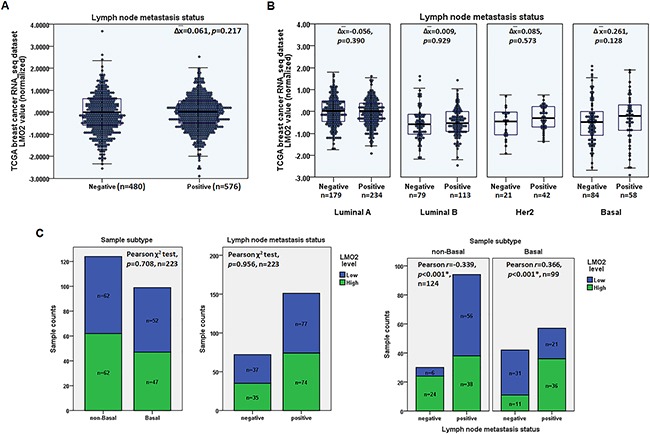
High LMO2 expression was positively associated with lymph node metastases in basal-type breast cancer **A**. 2D scatter plot and box plot showing the medians and distribution of LMO2 mRNA expression level in lymph node metastasis-negative and -positive samples in the TCGA breast invasive carcinoma RNA_seq dataset. Mean difference of LMO2 mRNA level (Δ), *p*-value from Student's *t*-tests, and sample count of each group are shown in the plot. **B**. 2D scatter plot and box plot showing medians and distribution of LMO2 mRNA expression level in lymph node metastasis-negative and -positive luminal A, luminal B, Her2, and basal-type breast cancer samples in the TCGA breast invasive carcinoma RNA_seq dataset. Data in each subgroup were analyzed using Student's *t*-tests; mean difference of LMO2 mRNA level in each subgroup (Δ), *p*-values, and sample count of each group are shown in the plot. **C**. Stacked bar plots showing the distribution of LMO2-high and -low expression samples in basal and non-basal groups, and in lymph node metastasis-negative and -positive groups of all, basal and non-basal breast cancer samples. *p*-values, Pearson *r* values, and sample count of each group are shown in the plots.

Using the Cancer Genome Atlas (TCGA) breast invasive carcinoma RNA_seq dataset containing 1,095 primary malignant tumor samples, the statistical analysis revealed no significant difference of the average LMO2 expression level between samples with and without lymph node metastasis (Student's *t*-test, *p*=0.217, Figure 1A). However, further analysis of the samples grouped by the PAM50 subtype revealed that, in basal-type breast cancers, LMO2 expression tended to be higher in samples with lymph node metastasis than in those without, although this difference did not reach statistical significance (Δ x¯=0.261, *p*=0.128, Figure 1B). In other subtypes, however, no differences of LMO2 expression were observed (Figure 1B). Furthermore, a sample set containing 223 clinically validated primary malignant tumors was subdivided into basal group including 99 samples characterized by the lack of estrogen receptor (ER), progesterone receptor (PR), and HER-2 expression in immunohistochemical evaluations [23], and non-basal group with the rest 124 samples. Based on anti-LMO2 immunohistochemistry (IHC) intensity (Supplementary Figure 1A), the statistical analysis showed that there was no significant difference of LMO2 expression between basal and non-basal groups, or between lymph node metastasis negative and positive groups in the overall sample set (Pearson χ^2^ test, Figure 1C, Supplementary Table 1). However, in basal group samples, high LMO2 expression was significantly positively correlated to lymph node metastasis (Pearson χ^2^ test, *p*<0.001, *r*=0.366, Figure 1C, Supplementary Table 1) and interestingly, LMO2 expression level showed a negative correlation with lymph node metastasis status in the non-basal group in this sample set (Pearson χ^2^ test, *p*<0.001, *r*=-0.339, Figure 1C, Supplementary Table 1). In addition, LMO2 levels as determined by IHC did not differ significantly between primary malignant tumors (n=237) and lymph node metastases (n=102) in the sample set (Pearson χ^2^ test, Supplementary Figure 1B, 1C, Supplementary Table 1), suggesting that no obvious LMO2 level alteration occurred after lymph node metastasis. These results suggest a potential function of LMO2 on promoting primary tumor cell metastasis specifically in basal-type breast cancer. Additionally, potential LMO2 functions on other breast cancer subtypes were also investigated in the TCGA dataset and interestingly, high LMO2 expression was found to predict a shorter overall survival in luminal A-type whereas a better outcome in Her2-type (Supplementary Figure 1D). These results further implicate the complicated and subtype-dependent functions of LMO2 on breast cancers.

### LMO2 promotes migration and invasion in basal-type breast cancer cells

**Figure 2:**
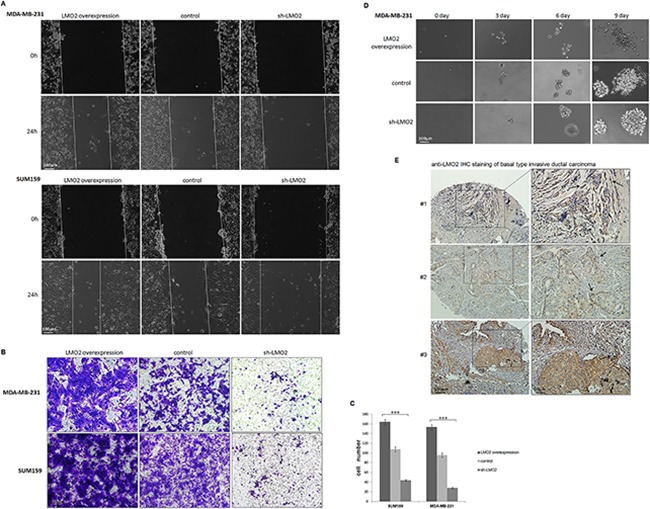
LMO2 promoted migration and invasion in basal-type breast cancer cells **A**. Images from the wound healing assay performed with LMO2 overexpression, control, and sh-LMO2 MDA-MB-231 and SUM159 cells 0 and 24 h after scratching. **B**. Images from Transwell invasion assay performed with MDA-MB-231 and SUM159 cells. Cells that passed through the Matrigel-coated membrane and attached to the underside of the membrane were stained with crystal violet and imaged with a light microscope. **C**. Quantification of Transwell invasion assay results. The bar plot shows the mean calculated cell numbers of three independent experiments; error bars indicate standard error. ***Student's *t*-test, *p*<0.001 compared to control. **D**. Images of the Matrigel growth assay with MDA-MB-231 cells. Colony morphologies of each cell strain 0, 3, 6, or 9 days after seeding are shown. **E**. Anti-LMO2 IHC stained images of basal-type breast cancer samples. Arrows indicate the invasive fronts of tumors where LMO2 staining was increased.

To further examine the cytological effects of LMO2 on breast cancers, a series of breast cancer cell lines, including Luminal, Her2 and basal subtype, with stable LMO2 overexpression or LMO2 knocking-down (sh-LMO2) were generated (Supplementary Figure 2A). In the wound-healing assay, overexpression of LMO2 increased, while knocking-down of LMO2 decreased, cell migration in basal-type breast cancer cell lines MDA-MB-231 and SUM159 (Figure 2A). However, LMO2 did not show any effect on cell migration in luminal A-type MCF-7 or Her2-type MDA-MB-435 cell lines (Supplementary Figure 2B). In a Transwell invasion assay, overexpression of LMO2 in MDA-MB-231 and SUM159 cells increased, while sh-LMO2 decreased, cell invasion (Figure 2B, 2C). Moreover, in a Matrigel-supported 3D cell culture, MDA-MB-231 cells overexpressing LMO2 formed more dispersed, loosely-organized colonies compared to control cells after as few as 3 days of culture, whilst sh-LMO2 cells formed more tightly attached, sphere-shaped colonies even after 9 days of culture (Figure 2D). Additionally, in many basal-type invasive breast cancer samples, LMO2 showed stronger staining at the edge of carcinoma nests, where cancer cells spread faster (Figure 2E, #1, #2), and at the invasive fronts of tumors (Figure 2E, #1, #3). Taken together, these results indicate a basal-type specific function of LMO2 on promoting breast cancer cell migration and invasion.

### LMO2 is predominantly located in the cytoplasm in breast cancer cells and affects actin cytoskeleton remodeling

**Figure 3:**
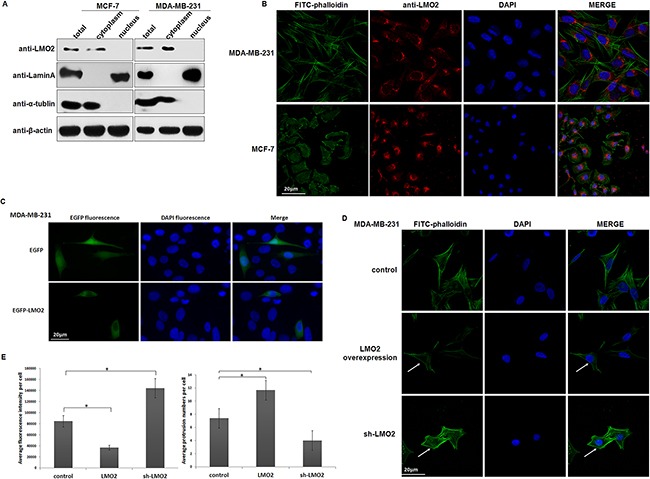
LMO2 was predominantly located in the cytoplasm in breast cancer cells and affected actin cytoskeleton remodeling **A**. Western blots of LMO2 expression in the total, cytoplasmic, and nuclear fractions of MDA-MB-231 and MCF-7 cells. β-actin, α-tubulin, and lamin A served as the loading control, cytoplasmic, and nuclear markers, respectively. **B**. Images of anti-LMO2 immunofluorescent staining showing the cytoplasmic localization of LMO2 in MDA-MB-231 and MCF-7 cells. Actin cytoskeleton was stained with FITC-phalloidin, LMO2 protein with an anti-LMO2 antibody and a Fluor-546 conjugated secondary antibody, and nuclei with DAPI. **C**. Images of MDA-MB-231 cells transiently expressing EGFP or EGFP-LMO2 fusion proteins. EGFP fluorescence revealed the subcellular localization of EGFP-LMO2 fusion protein or EGFP control. Nuclei were stained with DAPI. **D**. Images of immunofluorescent staining of the actin cytoskeleton with FITC-Phalloidin. Nuclei were stained with DAPI. Arrows indicate differences of actin cytoskeleton in LMO2 overexpression, control, and sh-LMO2 MDA-MB-231 cells. **E**. Bar plots of average fluorescence intensity and average cell protrusions in LMO2 overexpression, control, and sh-LMO2 MDA-MB-231 cells. Fluorescence values were evaluated in 10 different images for each experiment. The bar plot depicts mean fluorescence intensity values from three independent experiments; error bars indicate standard errors. *Student's *t*-test, *p<*0.05. Cell protrusions were counted in each cell in all images; bars represent mean protrusions/cell in 10 different images, and error bars indicate standard error. *Student's *t*-test, *p<*0.05.

Although LMO2 is traditionally recognized as a transcriptional factor, our data indicated a primarily cytoplasmic location of LMO2 in normal breast duct epithelia and breast cancer cells (Supplementary Figure 1A, 1B, Figure 2E). Immuno-blots of cytosolic and nuclear fractions from MDA-MB-231 and MCF-7 breast cancer cells revealed that LMO2 protein was much more abundant in the cytoplasm than in the nucleus (Figure 3A). Anti-LMO2 immunofluorescent staining of MDA-MB-231 and MCF-7 cells confirmed the predominantly cytosolic localization of LMO2, which was particularly enriched in the cytoplasmic space around the nucleus (Figure 3B). In contrast, in hematopoietic-derived K562 cells, consistent with previous reports, LMO2 was primarily located in the nucleus (Supplementary Figure 3A, 3B). Moreover, exogenous expression of EGFP-LMO2 fusion protein in MDA-MB-231 cells resulted in a primarily cytoplasmic green fluorescent signal, while the control EGFP signal appeared both in the cytoplasm and nucleus (Figure 3C).

Notably, the strength and total amounts of microfilaments (stress fibers) were significantly reduced in LMO2 overexpression cells, particularly in the cellular space around the nucleus where LMO2 was enriched; in contrast, increased microfilament formation, along with fewer cell protrusions and more attached cell morphologies, was observed in sh-LMO2 cells (Figure 3D, 3E). These results suggest that cytoplasmic LMO2 participates in the regulation of actin cytoskeleton remodeling and relatively high abundance of LMO2 causes microfilaments instability in basal-type breast cancer cells.

### LMO2 blocks the phosphorylation of cofilin1 by LIMK1 and reduces stress fiber formation in basal breast cancer cells

**Figure 4:**
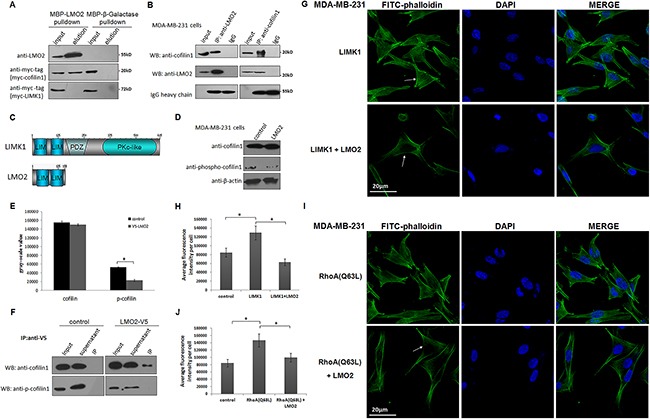
LMO2 blocked LIMK1-mediated cofilin1 phosphorylation and impaired stress fiber formation in basal breast cancer cells **A**. MBP-pulldown assay measuring binding between MBP-LMO2 recombinant fusion proteins and myc-tagged cofilin1/LIMK1 form HEK293T cell lysates. The MBP-β-Galactase fusion proteins were used as the negative control. **B**. Co-immunoprecipitation assay confirming interaction between endogenous cofilin1 and LMO2 in MDA-MB-231 cells. **C**. The structures of LMO2 and LIMK1. The similar tandem LIM domains of LMO2 and LIMK1 are depicted. **D**. Western blots of total cofilin1 and cofilin1 phosphorylated at Ser3 (p-cofilin1) in LMO2-overexpressing and control MDA-MB-231 cells. β-actin was used as the loading control. **E**. Quantification of gray-scale values of total cofilin1 and p-cofilin1 immunoblot bands in LMO2 overexpression and control MDA-MB-231 cells; bars represent the means of three replicates for each sample, and error bars indicate standard error. *Student's *t*-test, *p<*0.05. **F**. Western blots of co-immunoprecipitation of V5-tagged LMO2 and total cofilin1 or p-cofilin1. Wild-type MDA-MB-231 cells were used as negative controls. **G**. Images of FITC-phalloidin immunofluorescent staining of the actin cytoskeleton after LIMK1 overexpression in LMO2 overexpression or control MDA-MB-231 cells; arrows indicate differences of actin cytoskeleton. Nuclei were stained with DAPI. **H**. Bar plot showing average fluorescence intensity calculated as described in Figure 3E. Data are shown as means from three independent experiments; error bars indicate standard errors. *Student's *t*-test, *p<*0.05. **I**. Images of immunofluorescent staining of the actin cytoskeleton with FITC-phalloidin after forced expression of RhoA (Q63L) in LMO2 overexpression or control MDA-MB-231 cells and **J**. bar plots of average fluorescence intensity.

In stress fiber dynamic regulation, active cofilin1 severs the microfilament nearest the pointed ends, causing actin cytoskeleton depolymerization and actin monomer release, and LIMK1 eliminates cofilin1 activity by phosphorylating its Ser3 residue [24]. Interestingly, cofilin1 was initially identified as a candidate LMO2 binding partner in this study using yeast two-hybrid screening (Supplementary Figure 4A, 4B). The maltose Binding protein (MBP)-pulldown assay further revealed that binding occurred between LMO2 and cofilin1, but not between LMO2 and LIMK1 (Figure 4A). A subsequent endogenous co-immunoprecipitation assay in MDA-MB-231 cells confirmed the interaction between LMO2 and cofilin1 as well (Figure 4B). Furthermore, the LMO2 protein structure was composed of two tandem LIM domains (Figure 4C). Although full-length LMO2 could bind with cofilin1 (Supplementary Figure 5A, 5B), truncated forms of LMO2 with either LIM domain (LIM1: 1-87 aa or LIM2: 88-158 aa) did not show obvious binding ability (Supplementary Figure 5A, 5C). These results suggest that the two tandem LIM domains of LMO2 function as one module and are both necessary for LMO2-cofilin1 interaction.

LMO2 and LIMK1 are both members of the LIM protein superfamily and share more than 40% amino acid sequence homology in their LIM domains (Figure 4C). The LIM domains in LIMK1 mediate its recognition and interaction with cofilin1 [25, 26], so we hypothesized that binding between LMO2 and cofilin1 blocked the phosphorylation of cofilin1 by LIMK1. Indeed, overexpression of LMO2 in MDA-MB-231 cells decreased the phosphorylation level of cofilin1 (p-cofilin1, Figure 4D, 4E). LMO2 also reduced cofilin1 phosphorylation in cells overexpressing LIMK1, despite elevated background p-cofilin1 levels (Supplementary Figure 5D, 5E). Moreover, co-immunoprecipitation assays revealed that p-cofilin1 did not co-immunoprecipitate with LMO2, despite interactions between LMO2 and total cofilin1 (Figure 4F), further supporting the blocking effect of LMO2 on LIMK1-mediated phosphorylation of cofilin1. In immunocytofluorescent images, overexpression of LIMK1 in MDA-MB-231 cells increased stress fiber formation and caused tightly attached cell morphology. However, co-overexpression of LMO2 with LIMK1 largely eliminated this LIMK1-induced effect (Figure 4G, 4H). In addition, forced expression of the constitutively active form of the small GTPase RhoA (RhoA (Q63L)), which continuously activates LIMK1 upstream of the LIMK1-cofilin1 axis [25], similarly increased stress fiber formation and LMO2 also largely reversed this effect (Figure 4I, 4J).

### LMO2 expression level is negatively associated with cofilin1 phosphorylation level in basal-type breast cancer samples

**Figure 5:**
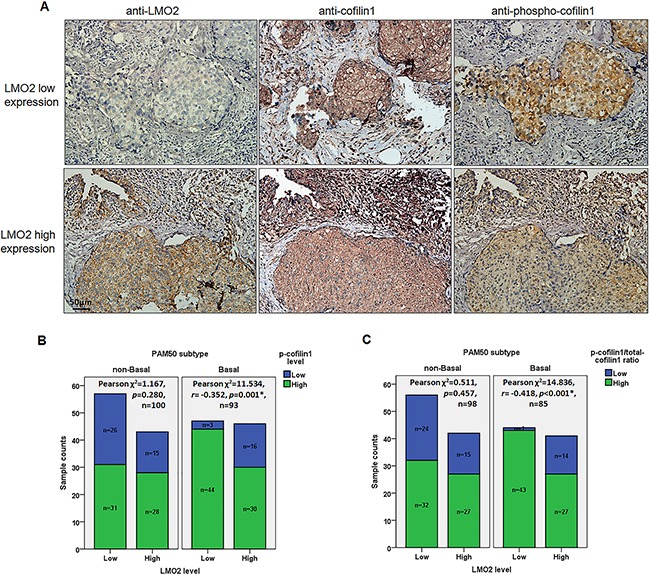
Phosphorylated cofilin1 level and p-cofilin1/total-cofilin1 ratio were negatively correlated with LMO2 level in basal-type breast cancer samples **A**. Representative images of IHC staining of LMO2, cofilin1, and p-cofilin1 in serial sections in a basal-type breast cancer sample set. LMO2 and p-cofilin1 levels were negatively correlated. **B**. Stacked bar plots showing the distribution of p-cofilin1-high and -low samples in LMO2-high and -low basal-type and non-basal-type breast cancer samples. *p*-values, Pearson *r* values, and sample counts are shown in the plots. **C**. Stacked bar plot showing p-cofilin1/total-cofilin1 ratio (R) distributions in LMO2-high and -low basal-type and non-basal-type breast cancer samples. P-cofilin1/total cofilin1 ratios (R) were used to evaluate relative levels of cofilin1 phosphorylation in different samples. Samples were divided into low (0≤R≤0.5) or high (0.5<R≤1) ratio groups; samples with R>1 were discarded. *p*-values, Pearson *r* value, and sample counts are shown in the plots.

Total cofilin1 generally showed stably high expression level in the clinical sample set (Figure 5A), and all breast cancer samples were subdivided into LMO2-low/high groups and p-cofilin1-low/high groups based on IHC scores. In the overall sample set, no statistical difference of p-cofilin1 level was observed between LMO2-low and LMO2-high groups (Pearson χ^2^ test, *p*>0.05, Supplementary Table 2). Notably, in basal-type breast cancer samples, p-cofilin1 level showed a significantly negative correlation with LMO2 expression level (Pearson χ^2^ test, *r*=-0.352, *p*=0.001, Figure 5B, Supplementary Table 2). In contrast, no statistical difference or correlations were observed in non-basal-type breast cancer samples (Pearson χ^2^ test, Figure 5B, Supplementary Table 2). To adjust the potential interference by the slight difference of total cofilin1 level in each sample on this analysis, the ratio of p-cofilin1 score to total cofilin1 score (marked as R) were calculated for each sample and all samples were further divided into low ratio (0≤R≤0.5) and high ratio (0.5<R≤1) groups. Again the statistical analysis revealed a negative correlation between LMO2 expression level and R values in basal-type samples (Pearson χ^2^ test, *r*=-0.418, *p*<0.001) but not in non-basal samples or the overall samples (Pearson χ^2^ test, Figure 5C, Supplementary Table 2). These results further support the basal-type specifically negative regulatory role of LMO2 on cofilin1 phosphorylation in breast cancer cells.

### LMO2 promoted *in vivo* invasiveness and distant metastasis of basal-type breast cancer cells in orthotopic xenograft SCID mice

**Figure 6:**
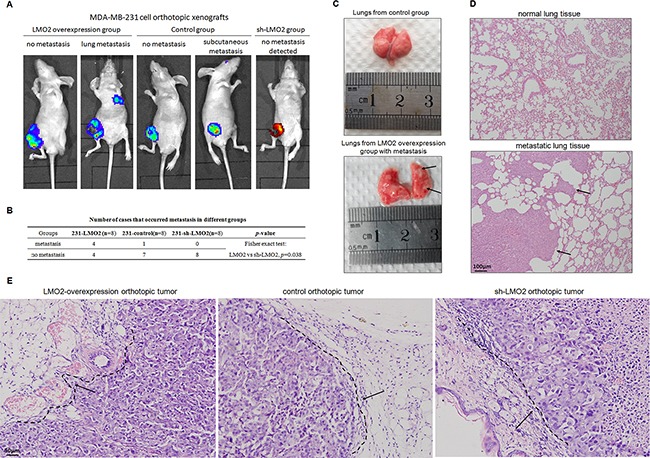
LMO2 promoted *in vivo* invasiveness and distant metastasis of basal-type breast cancer cells in orthotopic xenograft SCID mice **A**. Representative *in vivo* images of LMO2 overexpression, control, and sh-LMO2 MDA-MB-231 group mice. LMO2-overexpressing and control cells were labeled as indicated; orthotopic and metastatic tumor cells are identified by fluorescence. **B**. Numbers of cases with distant metastasis in the LMO2 overexpression, control, and sh-LMO2 groups. Data were analyzed using Fisher's exact test; *p*=0.038 for the LMO2 overexpression compared to the sh-LMO2 group. **C**. Representative images of lungs with and without metastases from mice in the LMO2 overexpression and control groups, respectively; arrows indicate metastases. **D**. H&E staining of normal tissue and lung metastases shown in (C); arrows indicate tumor cells. **E**. Representative H&E staining of orthotopic tumors in LMO2 overexpression, control, and sh-LMO2 group mice. Tumor cell and adjacent normal tissue boundaries are marked with a dotted line and indicated with arrows.

In orthotopic xenograft BALB/c SCID mouse models, tumor growth among the LMO2 overexpression, control, and sh-LMO2 groups after relevant MDA-MB-231 cell injection was monitored up to 8 weeks. Lung metastases were observed in 50% of LMO2 overexpression group mice (4 of 8), one subcutaneous metastasis near the eyelid was noted in a control mouse, and no metastases were found in the sh-LMO2 group mice using *in vivo* imaging method (Figure 6A, 6B). In addition, 2-5 lung metastases were found in the 4 metastatic mice in LMO2 overexpression group, while none were found in the other groups (Supplementary Figure 6). Images of lungs with metastases and control lungs are shown in Figure 6C, and H&E staining further confirmed tumor cell infiltration (Figure 6D). In addition, H&E staining of orthotopic tumors revealed that LMO2-overexpressing tumors were more aggressive and invaded adjacent tissues, while control and sh-LMO2 tumors were less aggressive and grew with relatively smooth, clear boundaries with adjacent tissue (Figure 6E). These results provide *in vivo* evidence for the LMO2 function on promoting tumor cell invasion and metastasis in basal-type breast cancer cells.

## DISCUSSION

The traditional recognition of LMO2 is that it initiates embryonic hematopoiesis and angiogenesis, and functions as a bridge molecule in the transcriptional complex in cell nucleus [26]. In this study, we reported that LMO2 was predominantly located in the cytoplasm in normal breast dust epithelia and breast cancer cells, and specifically increased basal-type breast cancer cell invasion and metastasis via blocking the LIMK1-mediated phosphorylation of cofilin1. Notably, no matter in nucleus or in cytoplasm, LMO2 generally exhibits complicated functions: as a transcriptional regulator, LMO2 performs dual-direction regulation patterns on different target genes via binding with different DNA-binding factors and binding on different DNA motifs [8–10]; as a cytoplasmic protein, LMO2 binds with a variety of proteins and participate in several cellular processes, either as an oncogene or as a tumor suppressor [14, 16, 27]. Herein we found that even in a certain cancer type, such as breast cancer, LMO2 functioned diversely in a subtype-dependent manner. These are possibly due to the LIM-domain only structure which can mediate a wide variety of protein-protein interactions [28]. This structural feature of LMO2 suggests that it could bind to diverse partners from multiple cellular pathways simultaneously and the predominant function of LMO2 in a certain cell type depends not only on the abundance of LMO2 itself but also on the abundance of LMO2 preferred partners, which can consume LMO2 molecules competitively and guide LMO2 to different function pathways.

Another interesting question is why LMO2 locates either in nucleus or in cytoplasm. LMO2 was initially expressed around E10.5 in mouse embryonic hemogenic endothelial cells (ECs) together with its known binding partners GATA2 and TAL1 [29], which are DNA-binding factors but did not express in most of the epithelia [26]. Our preliminary data in this study suggested that co-expression of LMO2 with GATA2 or TAL1 could help LMO2 nuclear translocation in some epithelia but not in all cases (Supplementary Figure 3C), and the regulatory patterns of LMO2 on several known target genes in hematopoietic-endothelial systems was also different from in epithelia (Supplementary Figure 3D, 3E) [9, 30, 31]. These may partially explain the phenomenon that LMO2 was predominantly located in nucleus in hematopoietic and endothelial cells but in cytoplasm in most of the epithelia.

Invasion and metastasis are hallmarks of highly aggressive tumor cells [32, 33], and are resulted primarily from enhanced cell migration controlled by actin cytoskeleton remodeling [34]. The LIMK1-cofilin1 axis is critical for coordinating cell motility and plays important roles in cancer cells. However, there were some puzzles for LIMK1 and cofilin1 expression patterns in cancers [35]: total cofilin1 is highly expressed in ovary, lung, breast, pancreatic, and renal cancer cells, while LIMK1 is also highly expressed in ovary, lung and breast cancers. However, phosphorylated cofilin1 levels are decreased in many cancer types. Herein our findings show that LMO2 directly blocks the LIMK1-mediated phosphorylation of cofilin1, suggesting that cytoplasmic LMO2 level could be an additional determinant for the phosphorylation level of cofilin1 in cancer cells, and loss of cofilin1 phosphorylation in various cancers could be the driven event to promote tumor cell invasion and metastasis.

Taken together, our study revealed a basal-type specific function of LMO2 on promoting tumor cell invasion and metastasis in breast cancers. In mechanism, cytoplasmic LMO2 blocks the LIMK1-mediated phosphorylation of cofilin1, causing actin cytoskeleton instability and enhanced tumor cell motility. These results provide novel evidence for the functional complexity of LMO2 in solid tumors and suggest the potential of LMO2 as a basal-type specific, metastasis-related biomarker for breast cancers on clinic.

## MATERIALS AND METHODS

### Online datasets and statistical analysis

The TCGA breast invasive carcinoma RNA_seq dataset (Level 3 data) was downloaded from the UCSC Cancer Genomics Browser (https://genome-cancer.ucsc.edu/). Processed LMO2 expression data and clinical information for each sample are described in the Supplementary files “TCGA_RNA_seq.xls”. Statistical analysis was performed with IBM SPSS Statistics version 20.0 (SPSS Inc., Chicago, IL).

### Clinical samples and immunohistochemistry

A total of 370 breast tissue samples, including 7 normal tissues, 24 benign tumors, 237 primary malignant tumors, and 102 lymph node metastases associated with their clinical information, were collected and arrayed by Alenabio Corporation (Xi’an, China) with the authorization of the local medical ethics committee. After tissue arrays were deparaffinized and rehydrated and antigen retrieval and blocking of endogenous peroxidases was performed, arrays were washed three times in 0.01 M PBS for 5 min each and blocked for 1 h in 0.01 M PBS supplemented with 0.5% Triton X-100 and 5% normal goat serum. Sections were then incubated with anti-LMO2, anti-cofilin1, or anti-phospho-cofilin1 antibodies (1:200) at 4°C overnight. After brief washing in 0.01 M PBS, sections were exposed to secondary antibody (1:500) for 1 hr at room temperature, washed, and incubated with DAB for 10 min. Finally, all sections were counterstained with hematoxylin. Each sample was scored from 0 to 5 based on cytosolic staining intensity by two pathologists who were blind to treatments. Samples with scoring discrepancies were re-evaluated and confirmed by a third observer.

### Plasmid constructs

The human LIMK1 and cofilin1 coding sequences were amplified, inserted into the pcDNA6B vector with a myc-tag, and confirmed by sequencing. Coding sequences of full-length LMO2 and its truncated forms were amplified and inserted into the pcDNA3.1/V5-His TOPO vector with a V5-tag (Invitrogen, Austin, TX) or the pEGFP-C3 vector with an EGFP-tag (BD Biosciences, San Jose, CA). The RhoA (Q63L) constitutively-active form expression vectors were kindly provided by Dr. Thomas J. Wandless of Stanford University. LMO2 expression and control lentiviral vectors (with a luciferase fluorescent marker), LMO2-shRNA lentiviral vector (with an mCherry fluorescent marker), and the Lenti-Pac™ HIV Expression Packaging Kit were purchased from GeneCopoeia (Rockville, MD). The HEK293T packaging cell line was used for lentiviral amplification according to GeneCopoeia's instructions.

### Cell culture and transfection

MDA-MB-231, SUM159, MDA-MB-435, MCF-7, HEK293T, and K562 cells were obtained from ATCC (University Boulevard, Manassas, VA) and cultured in RPMI 1640 or DMEM medium supplied with 10% FBS. Transfection of MDA-MB-231 and HEK293T cells was performed using Lipofectamine 2000 according to the manufacturer's instructions (Invitrogen, Austin, TX). LMO2 expression and control lentiviral vectors with the luciferase marker and LMO2-shRNA lentiviral vector with the mCherry marker were packaged for infection. Stable cell strains were selected and cultured in medium supplemented with 2 μg/mL puromycin three days after lentiviral infection. Cells transfected with RhoA (Q63L) expression vectors were cultured in medium supplemented with 500 μg/mL G418 for 1 week to remove non-transfected cells before assays were conducted.

### Wound-healing, transwell invasion, and matrigel growth assays

For the wound healing assay, cells were seeded into a 24-well plate (2×10^5^ cells/well) and gently scratched with a 200 μL pipette tip across the center of the well 24 h later. Images were captured 0, 12, and 24 h after scratching with a phase contrast microscope (Leica DC100, Germany). For the Transwell invasion assay, 6×10^4^ cells/well in RPMI1640 medium with 1% FBS were seeded in the upper 6.5mm Transwell chamber (Corning, Tewksbury, MA) previously coated with Matrigel (BD Biosciences, San Jose, CA) and placed in a 24-well plate with RPMI1640 medium containing 20% FBS. After 24 h of incubation, cells in the upper chamber were removed with a cotton swab and cells attached to the underside of the membrane were fixed with 4% formaldehyde, stained with 0.1% crystal violet, and either imaged under a microscope or eluted with 33% acetic acid and quantified at 570 nm. For the Matrigel growth assay, 1×10^3^ cells in 100 μL RPMI 1640 medium were mixed 1:1 with BD Matrigel and seeded in a 24-well plate at 37°C for 15 min to solidify the Matrigel; an additional 400 μL of RPMI was then added. Colony morphology in each well was imaged every 24 h for up to 9 days with a phase contrast microscope.

### Protein extraction, MBP-pulldown, and co-immunoprecipitation assays

The extraction of total, cytoplasmic, and nuclear protein was performed using a protein extraction kit (CWBIO, Beijing, China). Protein concentration was assayed with the BCA Protein Assay Kit (Pierce Biotechnology, Rockford, IL). One mg of total protein from each sample was used for MBP-pulldown or co-immunoprecipitation assays following previously described protocols [9]. Antibody information is listed in the supplementary information. Gray scale quantification of immunoblot bands was performed using ImageJ software.

### Immunocytofluorescence and confocal microscopy

First, 2×10^4^ cells were seeded on cell chamber slides (Corning, Tewksbury, MA) and placed in a 24-well plate 24 h prior to assays. Cells cultured on chamber slides (Corning, Tewksbury, MA) were fixed with 4.0% paraformaldehyde (PFA) at room temperature for 15 min. Slides were blocked with 2% BSA in PBST (PBS + 0.25% Triton X-100) and then incubated with antibodies (LMO2, cofilin1, Arp3, or profilin1, 1:200) overnight at 4°C. Slides were then washed with PBST three times after incubation with secondary antibodies (1: 500) at room temperature for 1 h. After washing, slides were incubated with DAPI (Invitrogen) at room temperature for 1 min. Images were obtained with a FV1000 confocal microscope (Olympus, Center Valley, PA). Quantification of fluorescence intensity was performed using ImageJ software.

### SCID mice xenograft experiments

Experimental protocols were approved by the Animal Care and Use Committee of the College of Lifesciences at Nankai University. BALB/c SCID mice (5 week-old females, 16-18 g) were injected in the right fat pad of the fourth mammary gland with 2×10^6^ cells (50 μL). Tumor growth was monitored every 5 days for up to 50 days, and tumor volume was calculated as follows: V(mm^3^)=a*b^2^/2, where *a* was the largest diameter and *b* was the perpendicular diameter. Xenograft mice were imaged 8 weeks after injection using an IVIS 200 Imaging System (Xenogen Corporation, Hopkinton, MA) according to equipment instructions. Orthotopic tumors and metastatic tissues were dissected, imaged, fixed with 4% formaldehyde, embedded in paraffin, sectioned at 5 μm, and used for H&E staining.

## SUPPLEMENTARY FIGURES AND TABLES




