# Supplementary material for: LMO2 promotes tumor cell invasion and metastasis in basal-type breast cancer by altering actin cytoskeleton remodeling
Source: Oncotarget. 2016 Nov 17;8(6):9513–24. doi: 10.18632/oncotarget.13434 (PMC5354749; doi:10.18632/oncotarget.13434)
Supplement: Supplementary file 1 [file oncotarget-08-9513-s001.pdf]

LMO2 promotes tumor cell invasion and metastasis in basal-type breast cancer by altering actin cytoskeleton remodeling

SUPPLEMENTARY METHODS

Yeast two-hybrid assay

The Matchmaker Gold Yeast Two-Hybrid system (Clontech, Palo Alto, CA) was used. The LMO2 coding sequence was inserted into the pGBKT7 vector to produce GAD4_BD-LMO2 fusion protein as bait. The pre-transformed library of human universal cDNA cloned into the pGADT7 vector was purchased from Clontech. Yeast strain mating and screening procedures were conducted according to the manufacturer's instructions. Positive clones (blue) were screened and re-seeded in a new selection medium plate, and each inserted cDNA fragment was PCR-amplified and sequenced. Potential LMO2 binding partners were confirmed using BLAST (NCBI).

Mammalian two-hybrid assay

The CheckMate Mammalian Two-Hybrid system (Promega, Madison, WI) was used. LMO2 (full-length), LIM1, and LIM2 coding sequences were subcloned into the pBIND vector, and the cofilin1 coding sequence was inserted into the pACT vector. pACT-cofilin1, pBIND-LMO2/-LIM1/-LIM2, and pG5luc reporter vectors were co-transfected into HEK293 cells at a ratio of 1:1:1 using Lipofectamine 2000. Luciferase activity was measured with a Dual-Luciferase Reporter Assay Kit (Promega) according to the manufacturer's instructions 24 h after transfection. pBIND-Id and pACT-MyoD vectors were used in combination as positive controls, and pBIND and pACT empty vectors were used as negative controls.

1. Primer information

Primer names	Sequences 5'- 3'	Restriction site
LMO2-forward	AATGCGGGTGAAAGACAAAG	
LMO2-reverse	CCCCAAAGTGCCTAAGAGTG	
GAPDH-forward	TGAAGGTCGGTGTGAACGGAT	
GAPDH-reverse	CATGTAGGCCATGA GGTCCACCAC	
E-LMO2 sense	GATG**AAGCTT**ATGTCCTCGGCCATCGAA	HindIII
Y-LOM2-antisense	CTT**GGATCC**CTATATCATCCCATTGATCTTAGTCC	BamH1
LMO2-antisense (V5)	TAGT**TCTAGA**CTTATCATCCCATTGATCTTAGTCC	XbaI
LIM1 antisense	TAGTTCTAGACTAAGCCTGAGATAGTCTCTCCGG	XbaI
LIM2 sense	GATGAAGCTTATGTCCTTTGGGCAAGACGGTCTCTG	HindIII
hLIMK1 sense	GATGAAGCTTATGAGGTTGACGCTACTTTGTTGCACC	HindIII
hLIMK1 antisense	TAGTTCTAGACTGTCGGGGACCTCAGGGTGGG	XbaI
hCofilin1 sense	GATGAAGCTTATGGCCTCCGGTGTGGCTGTC	HindIII
hCofilin1 antisense	TAGTTCTAGACTCAAAGGCTTGCCCTCCAGGGA	XbaI

2. Antibody information

Product Name	Source	Product Number	Company	Application *
**Myc-tag**	rabbit mAb	2278	CST	WB, IP
**V5-Probe(G-14):sc-83849**	rabbit pAb		Santa Cruz	WB, IP
**Anti-rabbit IgG**	HRP-linked antibody	7074	CST	WB
**Anti-mouse IgG**	HRP-linked antibody	7076	CST	WB
**Alpha-Tubulin**	rabbit mAb	ab108629	Abcam	WB
**β-actin Antibody**	rabbit pAb	21338	SAB	WB
**Profilin-1 Antibody**	rabbit pAb	3237	CST	WB, IP, IF
**GAL4(DBD):sc-577**	rabbit pAb		Santa Cruz	WB
**Myc-Tag (9B11) Mouse mAb**	Mouse mAb	2276	CST	WB, IF
**Anti-LMO2 antibody[EP3257]**	rabbit mAb	ab91652	Abcam	WB, IP
**Anti-Lamin A antibody**	rabbit pAb	ab26300	Abcam	WB
**Phospho-Cofilin (Ser)(77G2)**	rabbit mAb	3313	CST	WB, IP, IF, IHC
**Cofilin(D3F9)XP**	rabbit mAb	5175	CST	WB, IP, IF, IHC
**GFP(D5.1)XP**	rabbit mAb	2956	CST	WB
**Anti-Cofilin (phospho S3)antibody**	rabbit pAb	ab12866	Abcam	WB, IP, IF, IHC
**Anti-Arp3 antibody[FMS338]**	mouse mAb	ab49671	Abcam	WB, IP, IF, IHC
**Anti-LMO2 antibody[1A9-3B11]**	mouse mAb	ab81988	Abcam	IF, IHC
**Anti-LMO2 antibody-ChIP Grade**	rabbit pAb	ab72841	Abcam	WB, IP
**Alexa Fluor 546 donkey anti-rabbit IgG**		A10040	Invitrogen	IF
**Alexa Fluor 568 donkey anti-mouse IgG**		A10037	Invitrogen	IF

* abbreviations: WB, Western blot; IP, Immunoprecipitation; IF, Immunofluorescence; IHC, Immunohistochemistry

SUPPLEMENTARY FIGURES AND TABLES

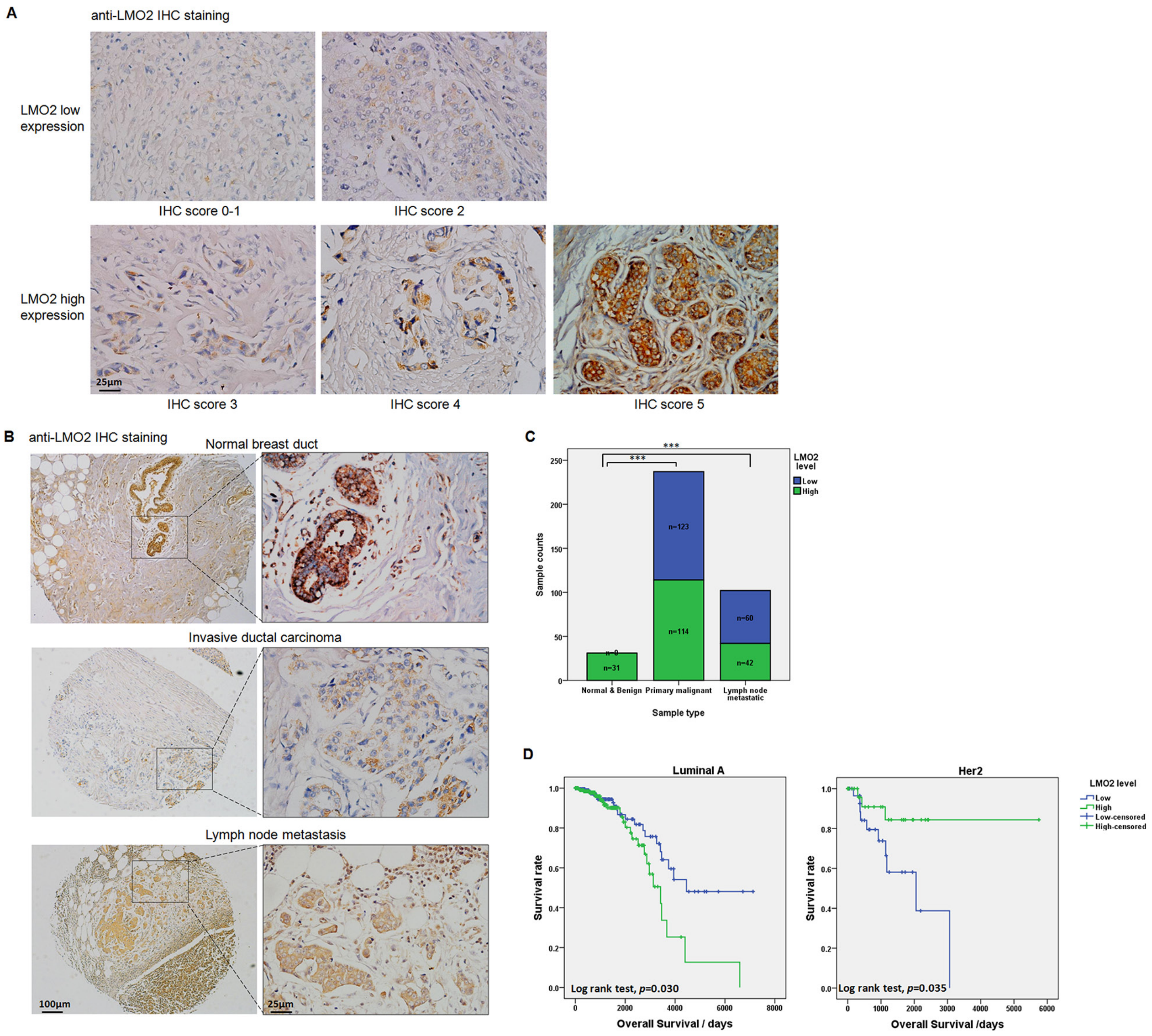

Supplementary Figure 1: Additional LMO2 expression features in online breast cancer RNA_seq dataset and 370 clinical patient samples. A. Representative images of breast tissue samples with anti-LMO2 IHC scores of 1-5. Samples with scores of 0-2 were designated low LMO2 expression, and samples with scores of 3-5 high LMO2 expression Anti-cofilin1 and anti-p-cofilin1 IHC staining were scored in the same samples using the same criteria. **B.** Representative images of anti-LMO2 immunohistochemical staining in normal breast tissue, primary invasive ductal carcinoma, and lymph node metastases from 370 clinical patient samples. LMO2 staining was predominantly cytoplasmic in most breast duct epithelia and invasive breast cancer cells. Nuclear LMO2 staining was observed in lymphocytes in the metastatic lymph node. **C.** Stacked bar plots showing distributions of LMO2-high and -low expression in 370 clinical samples in normal/benign group, primary malignant group, and lymph node metastasis group samples. Sample counts for each group are shown in the plots. ***Pearson χ^2 test, $p<0.001$. **D.** Kaplan-Meier curve of luminal A and Her2 type breast cancer patient survival. Survival data were obtained from the TCGA breast invasive carcinoma RNA_seq dataset. Patients were divided into high- and low-LMO2 expression groups based on the median expression of each subtype. Log-Rank test *p*-values are shown in each plot.

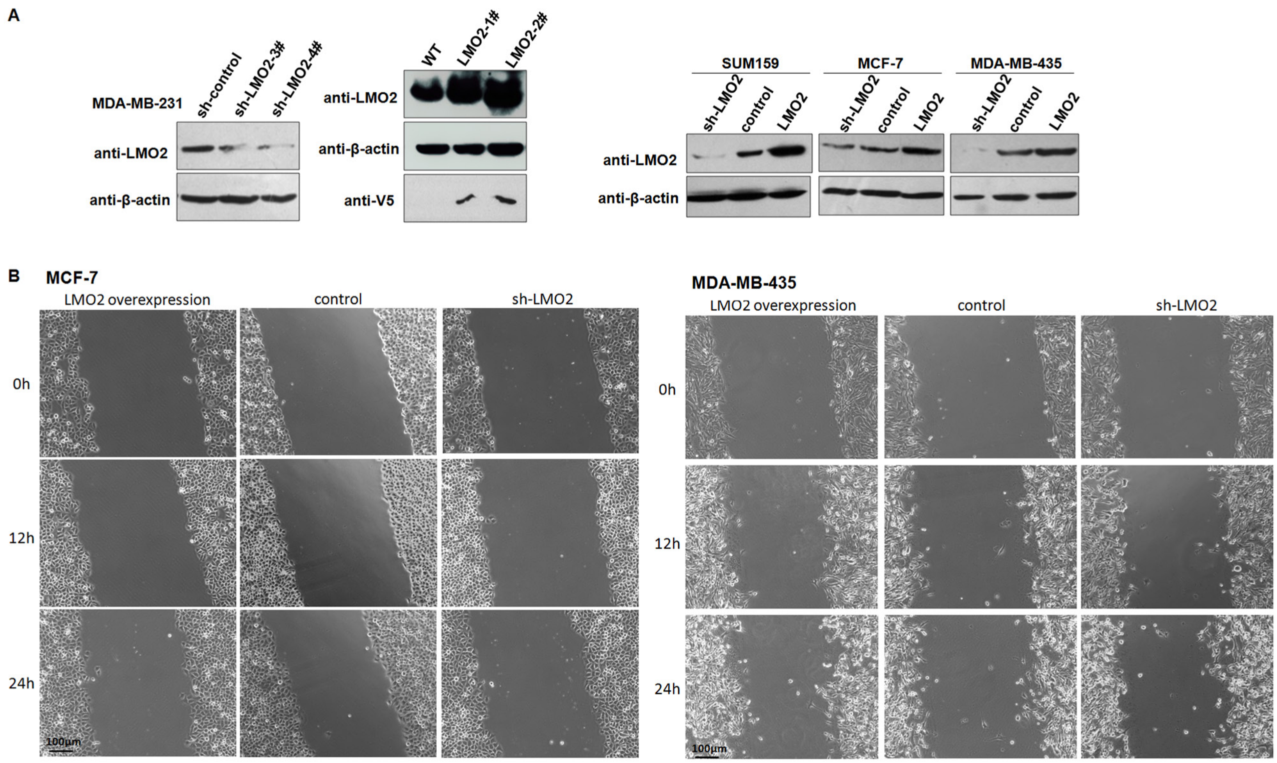

Supplementary Figure 2: Cytological effects of LMO2 in different breast cancer cell subtypes. A. Western blots confirmed overexpression of LMO2 and knockdown of endogenous LMO2 in lentivirally-infected, puromycin-selected MDA-MB-231, SUM159, MCF-7 and MDA-MB-435 breast cancer cells. LMO2 expression was measured using anti-LMO2antibody and anti-V5 tag antibody (for overexpression of LMO2 with a V5 tag in MDA-MB-231cells). β-actin was used as a loading control. **B.** Images from wound healing assays performed with LMO2 overexpression, control, and sh-LMO2 MCF-7 and MDA-MB-435 cells 0, 12, and 24 h after scratching.

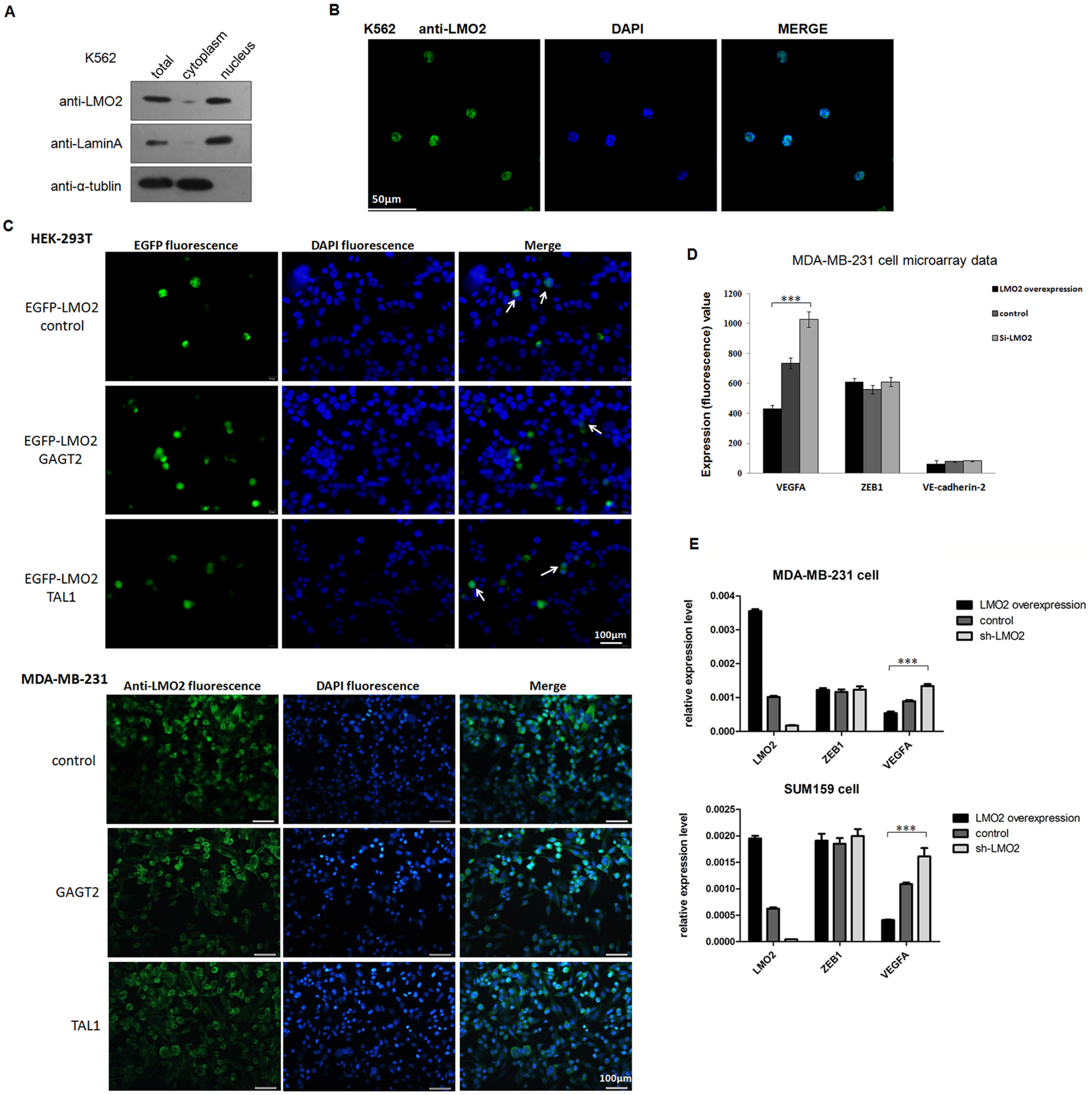

Supplementary Figure 3: LMO2 was located primarily in the nucleus in hematopoietic-derived K562 cells. A. Western blots showing LMO2 expression in the total, cytoplasmic, and nuclear fractions of K562 cells. α-tubulin and lamin A were used as cytoplasmic and nuclear markers, respectively. **B.** Immunofluorescent images showing the nuclear localization of LMO2 in K562 cells. LMO2 protein was stained with anti-LMO2 and Fluor-488-conjugated secondary antibodies. Nuclei were stained with DAPI. **C.** Images of EGFP fluorescence in HEK293T cells co-transfected with EGFP-LMO2 and control/GATA2/TAL1 vector and of anti-LMO2 immunofluorescent staining in MDA-MB-231 cells expressing GATA2/TAL1. EGFP and Fluor-488 fluorescence revealed the subcellular localization of endogenous LMO2 and EGFP-LMO2 fusion protein, respectively. Nuclei were stained with DAPI. **D.** Bar plot of ZEB1, VEGFA, and VE-cadherin microarray data from LMO2-overexpressing, control, and sh-LMO2 MDA-MB-231 cells. Error bars indicate standard error of three repeats for each sample. ***Student's*t*-test, p<0.001 compared to control. **E.** Bar plot of LMO2, ZEB1, and VEGFA Q-PCR expression data in LMO2-overexpressing, control, and sh-LMO2 MDA-MB-231 and SUM159 cells. Error bars indicate standard errors of three independent experiments for each sample. ***Student's *t*-test, p<0.001 compared to control.

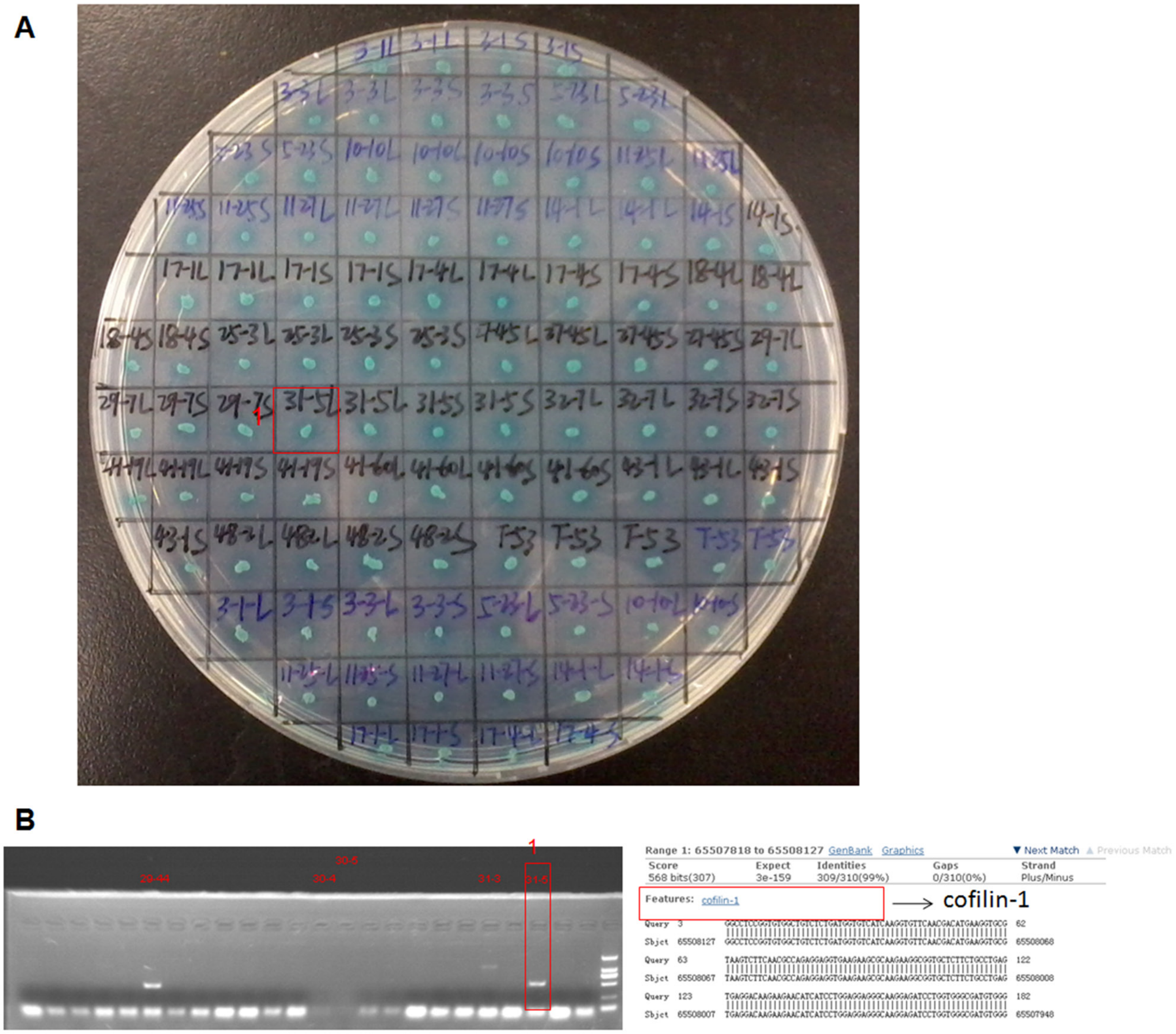

Supplementary Figure 4: Identification of cofilin1 as a LMO2 binding partner by yeast two-hybrid screening. A. Images of final selected positive yeast colonies that contained gene fragments of potential LMO2 binding partners after mating and stringent selection. **B.** Images of PCR-amplified gene fragments from selected positive colonies inserted into the pGADT7 vector. Amplified DNA fragments were sequenced and then aligned using BLAST. Cofilin1, was screened using this procedure.

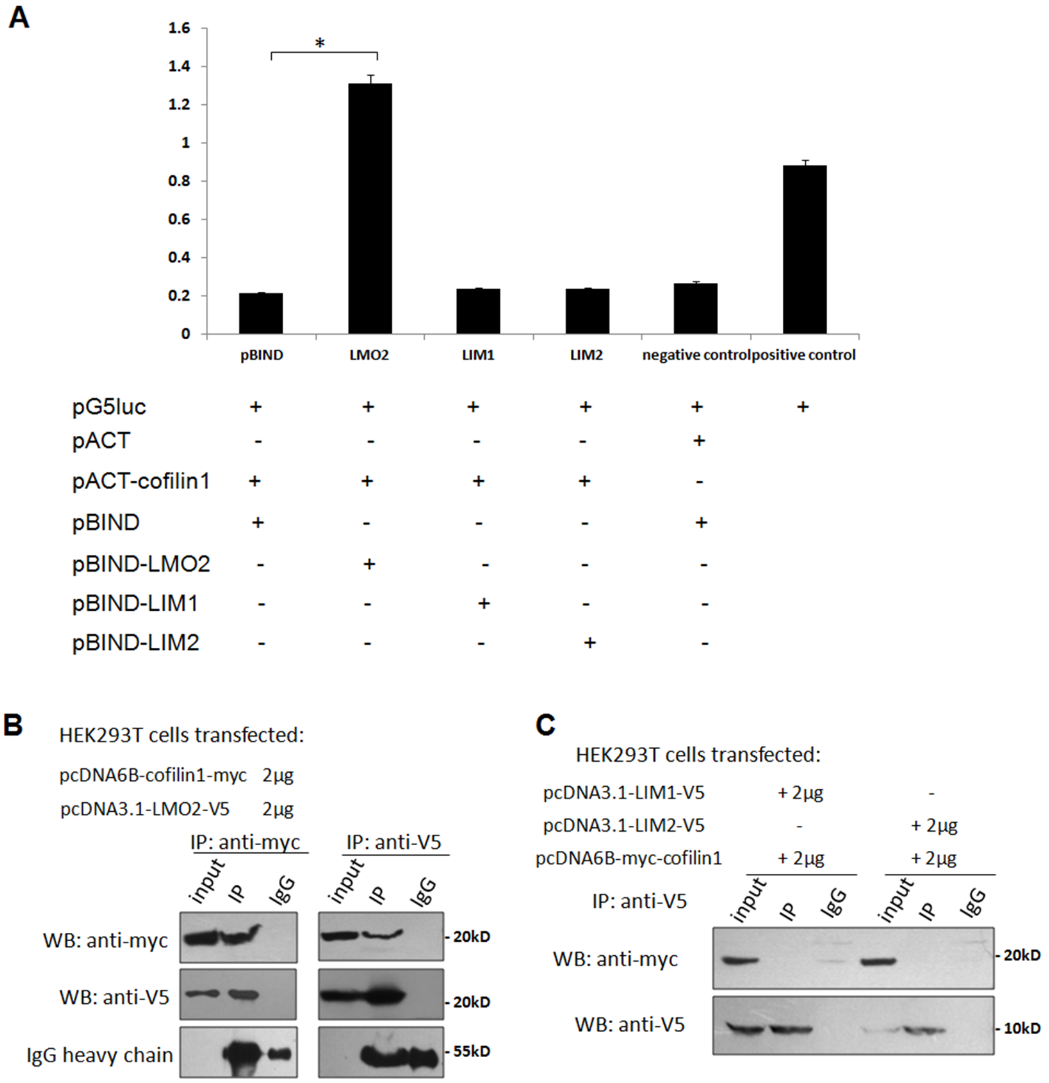

Supplementary Figure 5: Individual LMO2 LIM domains did not interact with cofilin1, and LMO2 reduced cofilin1 phosphorylation in MDA-MB-231 LIMK1-overexpressing cells. A. Bar plot of the mammalian two-hybrid assay. Cofilin1 interacted with full-length LMO2, but not with the truncated form of LMO2. pACT, activation domain fusion protein expression plasmid; pBIND, GAL4 binding domain fusion protein expression plasmid. Plasmids that were co-transfected into HEK293 cells are marked. Relative *luciferase* activity measured 24 h after transfection indicated the intensity of interactions between fusion proteins. Bars represent the means of three independent experiments; error bars indicate standard error. *Student's *t*-test, $p<0.05$. **B.** Western blot images of co-immunoprecipitation assays in HEK293T cells transiently co-transfected with V5-LMO2 and myc-cofilin1 vectors. **C.** Western blot images of the co-immunoprecipitation assay in HEK293T cells transiently co-transfected with V5-tagged truncated forms of LMO2 and myc-cofilin1 vectors. (*Continued*)

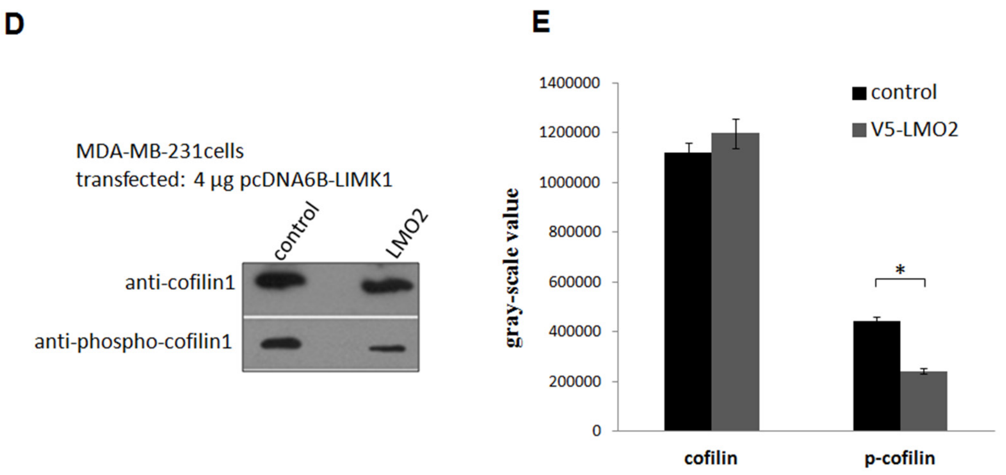

Supplementary Figure 5: (*Continued*) Individual LMO2 LIM domains did not interact with cofilin1, and LMO2 reduced cofilin1 phosphorylation in MDA-MB-231 LIMK1-overexpressing cells. D. Western blots of total cofilin1 and cofilin1 phosphorylated at Ser3 (p-cofilin1) in LMO2-overexpressing and control MDA-MB-231 cells also overexpressing LIMK1. **E.** Gray-scale quantification of total cofilin1 and p-cofilin1 immunoblot bands. Bars represent the means of three replicates for each sample; error bars indicate standard errors. *Student's *t*-test, $p<0.05$.

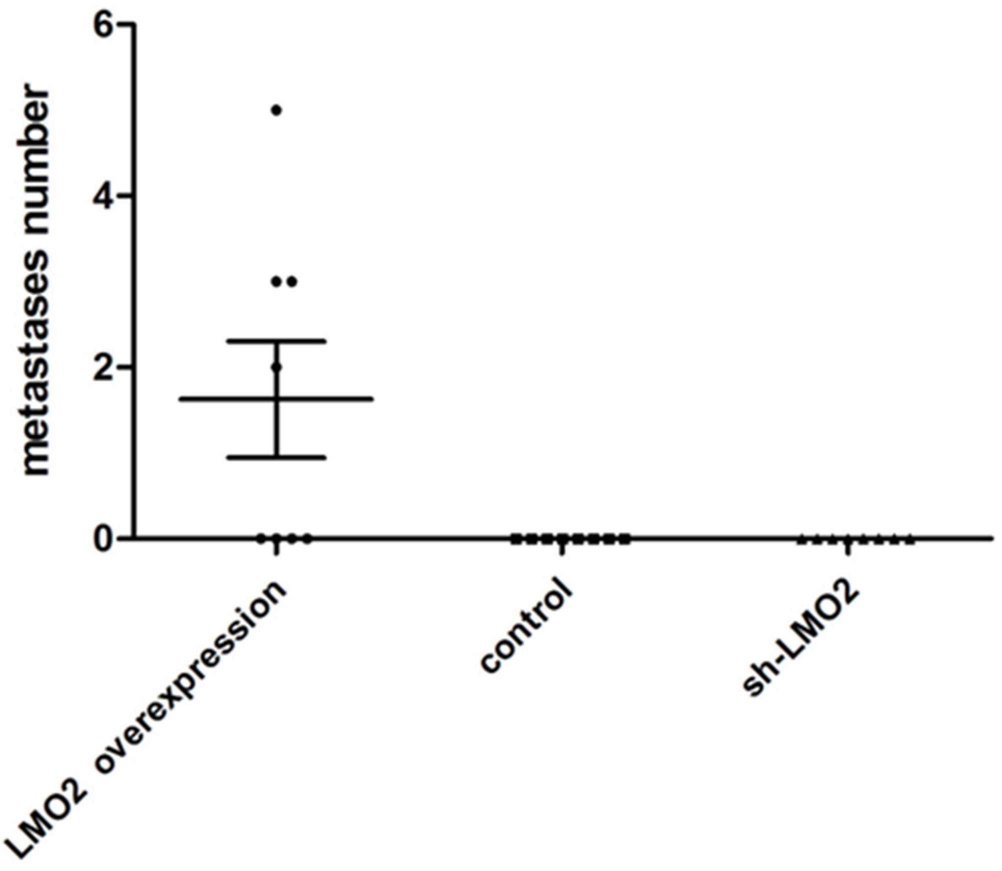

Supplementary Figure 6: Metastases of LMO2 overexpression, control and sh-LMO2 MDA-MB-231 cells in orthotopic xenograft SCID mice. Scatter plot of metastases in lungs from LMO2 overexpression, control and sh-LMO2 group mice was shown.

Supplementary Table 1: Summary of LMO2 expression based on sample type, PAM50 subtype, and lymph node metastasis status in clinical breast cancer samples

Clinical characteristic	LMO2 expression			Pearson χ^2	*p*-value
	Low	High	n=		
Sample types	183	187	370	33.406 (total)	$p<0.001$*
Normal & Benign (1)	0	31	31	28.656(1vs2)	$p<0.001$*
Primary malignant (2)	123	114	237	32.353 (1vs3)	$p<0.001$*
Lymph node metastasis (3)	60	42	102	1.466 (2vs3)	$p=0.226$
PAM50 subtypes	114	109	223		
Basal	52	47	99	0.140	$p=0.708$
Non-basal	62	62	124		
Lymph node metastasis status					
All primary malignant samples	114	109	223		
Negative	37	35	72	0.003	$p=0.956$
Positive	77	74	151		
Basal-type samples	52	47	99		
Negative	31	11	42	13.252	$p<0.001$*
Positive	21	36	57	Pearson $r=0.366$	
Non-basal-type samples	62	62	124		
Negative	6	24	30	14.274	$p<0.001$*
Positive	56	38	94	Pearson $r=-0.339$	

* Statistically significant

Supplementary Table 2: Summary of correlations between p-cofilin1 and p-cofilin1/total-cofilin1 ratios and LMO2 expression in different clinical breast cancer sample subtypes

Clinical characteristic	LMO2 expression			Pearson χ^2	*p*-value
	Low	High	n=		
All primary malignant samples	114	109	223		
p-cofilin1	104	89	193	1.080	$p= 0.299$
Low	29	31	60		
High	75	58	133		
p-cofilin1/total cofilin1 ratio (R)	100	83	183	2.154	$p= 0.142$
Low (0≤R≤0.5)	25	29	54		
High(0.5<R≤1)	75	54	129		
Basal-type samples					
p-cofilin1	47	46	93		
Low	3	16	19	11.534	$p= 0.001$*
High	44	30	74	Pearson $r= -0.352$	
p-cofilin1/total cofilin1 ratio (R)	44	41	85		
Low (0≤R≤0.5)	1	14	15	14.836	$p< 0.001$*
High(0.5<R≤1)	43	27	70	Pearson $r= -0.418$	
Non-basal-type samples					
p-cofilin1	57	43	100	1.167	$p= 0.280$
Low	26	15	41		
High	31	28	59		
p-cofilin1/total cofilin1 ratio (R)	56	42	98	0.511	$p= 0.475$
Low (0≤R≤0.5)	24	15	39		
High(0.5<R≤1)	32	27	59		

* Statistically significant

Supplementary File 1: “TCGA_RNA_seq.xls”.
